# Phosphorylation of inner core heptose is a major determinant of bacterial surface lipopolysaccharide recognition by the innate immune protein hSP-D

**DOI:** 10.1016/j.jbc.2026.111307

**Published:** 2026-02-20

**Authors:** Harry M. Williams, Alastair Watson, Jens Madsen, Howard W. Clark, Derek W. Hood, Stefan Oscarson, Trevor J. Greenhough, Annette K. Shrive

**Affiliations:** 1 School of Life Sciences, Keele University, Staffordshire, UK; 2 Division Faculty of Medicine, Department of Child Health, University of Southampton, Hampshire, UK; 3 School of Clinical Medicine, University of Cambridge, Cambridge, UK; 4 Targeted Lung Immunotherapy Group, Neonatology, The EGA Institute for Women's Health, University College London, London, UK; 5 Mammalian Genetics Unit, MRC Harwell Institute, Harwell Science and Innovation Campus, Oxfordshire, UK; 6 Centre for Synthesis and Chemical Biology, University College Dublin, Belfield, Dublin, Ireland

**Keywords:** surfactant protein D, crystal structure, lipopolysaccharide-binding protein, innate immunity, host–pathogen interaction, structural biology

## Abstract

The innate immune protein human surfactant protein D (SP-D) recognizes pathogens in the lungs *via* binding to carbohydrate surface structures. SP-D targets gram-negative bacterial lipopolysaccharide *via* calcium-dependent binding, preferentially to the inner core heptose (HepI). To further investigate this recognition, we have determined the high-resolution crystal structures of a trimeric recombinant fragment of human SP-D complexed with synthetic di-saccharide and trisaccharides, HepI-Kdo, HepIII-HepII-HepI, and HepII-HepI phosphorylated at either HepI or HepII, inner core lipopolysaccharide motifs common to many gram-negative bacteria. In contrast to acid-hydrolyzed lipopolysaccharide used in several previous studies, these synthetic saccharides allow the presentation of both the innermost Kdo in its natural pyranose form and heptose phosphorylation. The structures confirm the flexibility of SP-D to adopt alternative binding modes when the preferred epitope is not available, reveal a preference for recognition of the reducing terminal heptose (HepI) *via* the glyceryl group, indicate that a single Kdo attached to HepI does not have a significant role in ligand recognition, and provide evidence that heptose phosphorylation is a major determinant of recognition. The disaccharide with HepII O4′ phosphorylation binds *via* the preferred HepI glyceryl-hydroxyls, while HepI O4′ phosphorylation reveals HepII binding *via* the pyranose ring O3′ and O4′ hydroxyls, which would not be possible with the usual HepII O3′ link to the outer core. The ability of HepI O4′ phosphorylation to prevent preferred HepI recognition suggests a role for heptose phosphorylation in shielding the bacterial LPS inner core from immune recognition.

Surfactant protein D (SP-D) is a collectin with a key role in the innate immune defense that is expressed in pulmonary, as well as nonpulmonary epithelia (1, 2, 3, 4, 5). Human SP-D (hSP-D) is known to exert significant antimicrobial effects principally *via* opsonization (6, 7, 8, 9) while also having a role in modulating the broader host response to pathogens by interacting with various cell-based receptors (10) including the osteoclast-associated receptor, also known as OSCAR (11), DC-SIGN (12), and natural killer cell membrane receptor, NKp46 (13).

The recognition of bacterial, fungal, and viral pathogens by hSP-D is mediated by the carbohydrate recognition domain (CRD), which detects carbohydrate residues embedded within the surface structures of pathogens. For gram-negative bacteria, this is achieved through calcium-dependent binding of bacterial lipopolysaccharide (LPS) carbohydrate *via* a conserved hydroxyl motif. Bacterial LPS is typically composed of three major domains: the lipid A domain, core oligosaccharide, and hypervariable O-polysaccharide (14). The core oligosaccharide itself can be further split into an inner and outer core. The inner core is composed of up to three *L*-glycero-α-*D*-manno-heptose (Hep) residues, one of which is attached to a core 3-deoxy-*D*-manno-oct-2-ulosonic acid (Kdo) residue (15) which may also have additional linked Kdo residues. The outer core is a nonrepeating polysaccharide consisting of hexose residues attached to the heptosyl backbone (14). hSP-D recognizes bacterial LPS through association with heptose and the LPS inner core (16, 17, 18), and this interaction varies with length, complexity, and composition of the LPS.

A trimeric recombinant fragment of hSP-D (rfhSP-D) which has anti-inflammatory activity in murine models of inflammation (19, 20, 21) has provided key insights into bacterial recognition by hSP-D. The gram-negative bacteria *Haemophilus influenzae* and *Salmonella enterica* are of particular interest because while they can precipitate serious illness in both children and immune-compromised adults, they are also both recognized and neutralized by hSP-D (22, 23). For example, *H. influenzae* serotype b strains are associated with invasive bacterial infections including meningitis and septicemia (24, 25), whereas acapsular or nontypeable strains of *H. influenzae* can cause sinusitis, conjunctivitis, otitis media, and acute lower respiratory tract infections (22). *S. enterica* is a pathogen that causes periodic outbreaks of gastroenteritis with some strains, such as *S. enterica* serovar (sv) Typhi, causing enteric fever (26).

Crystallographic analyses have revealed how hSP-D interacts with, and recognizes, a range of carbohydrate structures from relatively simple carbohydrates (17, 27, 28, 29, 30), to more complex structures, such as the purified core oligosaccharide from rough strains of the gram-negative bacteria *H. influenzae* and *S. enterica* (18, 31). In each case, recognition of carbohydrate by hSP-D is achieved through calcium-dependent binding, at the primary calcium site, of a mannose-type or stereochemically-equivalent equatorial hydroxyl pair such as O6′, O7′ of a core heptose or O3′, O4′ of a terminal glucose, with the binding pocket flanking residues Arg343 and Asp325 contributing to recognition and binding (18, 27, 30, 31, 32). The importance and influence of these binding-site flanking residues in both ligand and pathogen recognition has been demonstrated not only through these extensive crystallographic analyses, but also through mutational studies (17, 29, 32). The high-resolution structures published by Clark *et al.* (18) and Littlejohn *et al.* (31) clearly demonstrated that hSP-D specifically and preferentially targets the bacterial LPS inner core of *H. influenzae* Eagan and *S. enterica* sv Minnesota rough strains R5 and R7 (rough mutant chemotypes Rc and Rd1) *via* the innermost heptose residue. These ligand-bound structures also highlighted that hSP-D has the flexibility and versatility to recognize alternative LPS epitopes, for example, a terminal glucose O3′, O4′ pair, when the preferred core heptose is unavailable (31).

In order to separate the core oligosaccharide from lipid A for use in protein crystallization studies, bacterial LPS is hydrolyzed with acetic acid (33). While this efficiently separates the hydrophilic core oligosaccharide from the hydrophobic lipid A domain, which is not involved in hSP-D recognition (16, 34), acid hydrolysis often also cleaves not only functional groups such as phosphate attached to core oligosaccharide residues but also Kdo residues extending from the central core Kdo. The β-elimination of Kdo-linked phosphate leads to the reorganization of Kdo into a 5-membered furanoid derivative, otherwise known as an anhydro-Kdo (18, 35). Accordingly, it has not been possible *via* this route to visualize the interaction of rfhSP-D with an intact Kdo residue nor has it been possible to reliably establish the role in ligand recognition by rfhSP-D of functional groups such as phosphate which are attached to core oligosaccharide residues. Binding and modeling studies of the interaction of SP-D with synthetic LPS inner core structures (36), using glycan array and surface plasmon resonance measurements, included nonphosphorylated representative *H. influenzae* and *S. enterica* core LPS components including the *H. influenzae* inner core HepIII-(1,2)-HepII-(1,3)-HepI and the HepI-(1,5)-KdoI common to both bacteria. These studies suggested that the Kdo does not influence binding but that the outer core residues might affect the preferred binding *via* HepI (31).

The work presented here builds on our previous research by using repurposed synthetic di-saccharides and trisaccharides (37, 38, 39), which correspond to elements of the core oligosaccharides of many gram-negative bacteria including *H. influenzae* and *S. enterica,* to overcome the disadvantageous effects of acid hydrolysis on bacterial LPS purified from whole bacteria. As a result, this work has allowed us to explore structurally for the first time not only how rfhSP-D recognizes the intact HepI-Kdo element of the LPS core (36) but also how rfhSP-D interacts with variably phosphorylated inner core oligosaccharide fragments. Ultimately, this promotes a greater understanding of how proteins of the innate immune response, in this case hSP-D and the biologically and therapeutically active trimeric recombinant fragment rfhSP-D, can recognize and interact with clinically important human pathogens.

## Results

**Figure 1:**
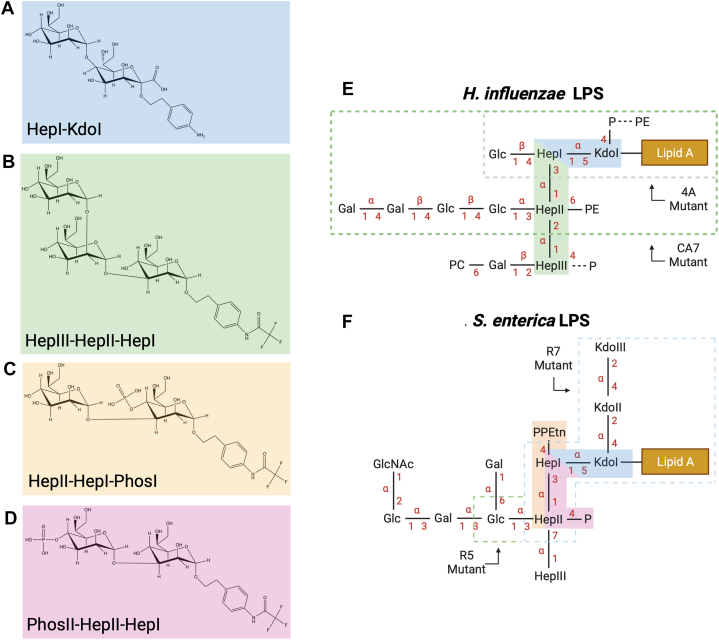
**Structure of the synthetic oligosaccharides corresponding to core elements of LPS from *Haemophilus influenzae* strain Eagan and *Salmonella enterica* sv Minnesota.***A*, the HepI-(1,5)-KdoI disaccharide [38; Supplementary Material] which maps on to the inner core of both *H. influenzae* and *S. enterica* LPS. *B*, the HepIII-(1,2)-HepII-(1,3)-HepI trisaccharide (39) which maps on to the *H. influenzae* LPS inner core (C) HepII-(1,3)-HepI-4-PhosI and (D) PhosII-4-HepII-(1,3)-HepI (37) which both map on to the *S. enterica* LPS inner core. *E*, the structure of LPS isolated from WT *H. influenzae* Eagan, adapted from (18) with 4A and CA7 referring to the rfaF and orfH mutant strains, respectively. *F*, the structure of the Ra phenotype LPS isolated from *S. enterica* rough mutant strains with the Rc(R5) and Rd1(R7) phenotypes indicated. LPS structures adapted from (42). Glc, glucose; GlcNAc, N-acetyl-glucosamine; Gal, galactose; Hep, L-D-Heptose; P, phosphate; PE/PEtn, phosphoethanolamine; PC, phosphocholine; Kdo, 3-deoxy-D-manno-oct-2-ulosonic acid. Chemical depictions of synthetic oligosaccharides produced in ChemDraw. Figure generated using BioRender.

Four synthetic oligosaccharides based on inner core motifs of *H. influenzae* Eagan and *S. enterica* sv Minnesota rough strains LPS were used for soaking into native crystals of rfhSP-D (see Fig. 1). These oligosaccharides all contain the inner core heptose that has been shown to be preferentially bound to hSP-D and also include a spacer on the reducing terminal sugar, replacing as appropriate where the heptose-linked inner core Kdo or lipid A would be attached. Three of these oligosaccharides, the triheptoside structure (HepIII-(1,2)-HepII-(1,3)-HepI) (39) and the two phosphorylated disaccharides (HepII-(1,3)-HepI-4-PhosI and PhosII-4-HepII-(1,3)-HepI) (37) include a p-trifluoroacetamidophenylethyl spacer while the Hep-Kdo disaccharide (HepI-(1,5)-KdoI) [38; Supplementary Material], common to both bacteria, includes a p-aminophenylethyl analog (see Fig. 1).

Crystal structures of rfhSP-D in complex with each of the synthetic oligosaccharides have been determined to high resolution, as follows: HepI-(1,5)-KdoI (1.75 Å), HepIII-(1,2)-HepII-(1,3)-HepI (1.63 Å), HepII-(1,3)-HepI-4-PhosI (1.92 Å), and PhosII-4-HepII-(1,3)-HepI (1.85 Å). The structures reveal ligand bound at the Ca1-binding pocket in two subunits (B and C) of the rfhSP-D trimer (Fig. S1) and no bound ligand in the third subunit (subunit A), consistent with the tight crystal packing around the ligand-binding pocket, as previously reported (18). Protein and ligand-calcium distances for each of the structures are provided in Table 1 and Table S1.

**Table 1:** Ligand (L)/water (W)–calcium/protein bond lengths (Å)

Atom 1		Atom 2		HepI-Kdo			HepIII-HepII-HepI			PhosII-HepII-HepI			HepII-HepI-PhosI		
				A	B	C	A	B	C	A	B	C	A	B	C
Ligand/water–calcium interactions															
HepI O6′ (L)	/W	Ca1		W/2.48	L/2.38	L/2.56	W/2.44	L/2.46	L/2.38	W/2.54	L/2.47	L/2.54	W/2.48	-	-
HepI O7′ (L)	/W	Ca1		W/2.56	L/2.39	L/2.53	W/2.56	L/2.55	L/2.40	W/2.55	L/2.53	L/2.43	W/2.56	-	-
HepII O3′ (L)	/W	Ca1		-	-	-	-	-	-	-	-	-	-	L/2.37	L/2.37
HepII O4′ (L)	/W	Ca1		-	-	-	-	-	-	-	-	-	-	L/2.34	L/2.38
Ligand–protein interactions															
HepI	O6′	Glu321	OE2	-	2.57	2.48	-	2.55	2.61	-	2.53	2.59	-	-	-
		Asn323	ND2	-	2.96	2.89	-	2.93	3.02	-	2.87	2.95	-	-	-
	O7′	Glu329	OE2	-	2.60	2.53	-	2.61	2.61	-	2.54	2.56	-	-	-
		Asn341	ND2	-	2.99	2.97	-	2.94	2.93	-	3.02	2.93	-	-	-
HepII	O3′	Glu329	OE2	-	-	-	-	-	-	-	-	-	-	2.61	2.54
		Asn341	ND2	-	-	-	-	-	-	-	-	-	-	3.02	2.99
	O4′	Glu321	OE2	-	-	-	-	-	-	-	-	-	-	2.55	2.49
		Asn323	ND2	-	-	-	-	-	-	-	-	-	-	2.91	3.03
HepI	O2′	Asp325	OD2	-	-	-	-	-	-	-	-	-	-	2.63	2.49
HepII	O2′	Arg343	NE	-	-	-	-	-	-	-	-	-	-	3.13	3.18
Phosphate	O1	Arg343	NH2	-	-	-	-	-	-	-	-	-	-	2.88	2.94
	O2	Arg343	NH1	-	-	-	-	-	-	-	-	-	-	-	3.08

**Figure 2:**
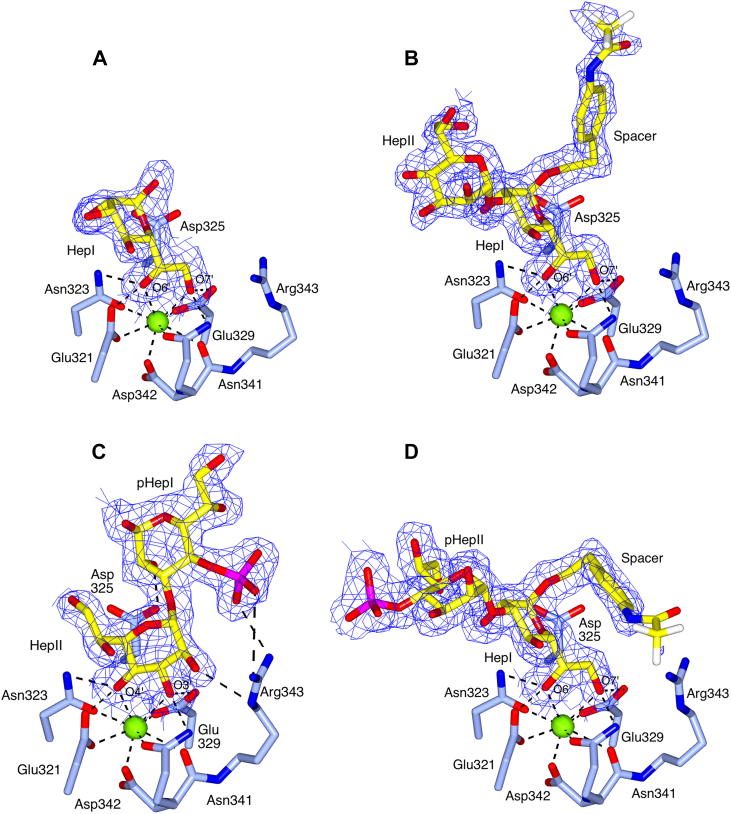
**Coordination of the synthetic saccharides in the rfhSP-D subunit B Ca1-binding pocket.** In each case, the ligand (*yellow*) is coordinated to Ca1 (*green sphere*) with key interactions (*dashed* lines) and binding site residues (*ice blue*) indicated. The 2mFo-DFc electron density map (*blue*) is clipped to the bound ligand and contoured at 1σ. *A*, HepI-(1,5)-KdoI. Only HepI, coordinated to Ca1 by the O6′ and O7′ hydroxyls, is visible in the electron density. *B*, HepIII-(1,2)-HepII-(1,3)-HepI with HepI, HepII and the spacer visible in the electron density and coordination to Ca1 *via* the HepI O6′ and O7′ hydroxyls. *C*, HepII-(1,3)-HepI-4-PhosI showing the alternative mode of recognition *via* the HepII O3′ and O4′ hydroxyls of HepII. The spacer linked to HepI is not visible in the electron density. *D*, PhosII-4-HepII-(1,3)-HepI, coordinated to Ca1 by the HepI O6′ and O7′ hydroxyls. Figure created using CCP4mg.

The extent to which each ligand can be modeled into the electron density varies between structures due to the nature and extent of contact and interaction with the protein. In the HepI-(1,5)-KdoI bound structure, HepI is clearly defined in the electron density, but there is no evidence to support the fitting of the Kdo residue or the spacer (Fig. 2*A*). For the HepIII-(1,2)-HepII-(1,3)-HepI bound structure (Fig. 2*B*), clear electron density is present for HepI–Hep II and for the HepI-linked spacer in subunit B, but not for HepIII. For the phosphorylated ligands corresponding to *S. enterica* core components, the complete HepII-(1,3)-HepI-4-PhosI bound ligand, except for the spacer, is fully defined in both subunits (Fig. 2*C*), while the whole of the PhosII-4-HepII-(1,3)-HepI ligand can be fitted into the subunit B electron density (Fig. 2*D*). In subunit C, there is only electron density present for HepI. Diagrams showing each of the protein–ligand interactions are in Fig. S2.

Recognition of the HepI-(1,5)-KdoI, HepIII-(1,2)-HepII-(1,3)-HepI, and PhosII-4-HepII-(1,3)-HepI ligands is achieved by Ca1 coordination of HepI O6′ and O7′ side chain hydroxyls (Table 1; Fig. 2), while recognition of the HepII-(1,3)-HepI-4-PhosI ligand is achieved by Ca1 coordination of the HepII O3′ and O4′ ring hydroxyls (Table 1; Fig. 2*C*). In all cases, the ligands are also coordinated by the protein through these hydroxyl pairs (17, 27), by Glu321, Asn323, Glu329, and Asn341. For the HepII-(1,3)-HepI-4-PhosI ligand, there are additional protein–ligand interactions (Table 1; Fig. 2*C*), between Arg343 and both the HepII O2′ hydroxyl (NE, 3.13, 3.18 Å) and the HepI-linked phosphate (NH1, 3.08 Å; NH2, 2.88, 2.94 Å), and between Asp325 OD2 and HepI O2′ (2.49, 2.63 Å). In each structure, ligand coordination is supported by an extensive network of water-mediated contacts and some limited interactions with residues in symmetry-related molecules (Table S2).

**Figure 3:**
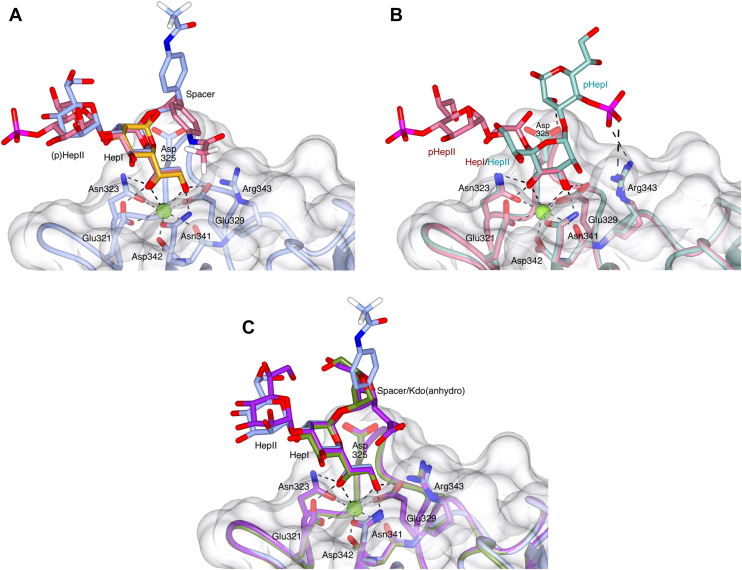
**Overlays of the ligand-bound structures and structures with bound anhydro Kdo.** All overlays generated by a least-squares fit of subunit B main-chain atoms, with key interactions (*dashed* lines) and binding site residues, including Arg343 and Asp325, indicated. *A*, HepI-(1,5)-KdoI (*gold*, only HepI visible in the electron density), HepIII-(1,2)-HepII-(1,3)-HepI-spacer (*ice blue*, HepIII not visible in the electron density), and PhosII-4-HepII-(1,3)-HepI-spacer (*pale red*). The two conformations of the HepI-linked spacer can be seen. The protein surface, main chain, and side chains are of the HepIII-(1,2)-HepII-(1,3)-HepI bound structure. *B*, the two phosphate-substituted diheptosyl ligands showing the different binding modes and orientations of the two ligands. PhosII-4-HepII-(1,3)-HepI (*pale red*, HepI-linked spacer omitted for clarity) coordinated to Ca1 by the HepI O6′ and O7′ hydroxyls and HepII-(1,3)-HepI-4-PhosI (*dark cyan*, HepI-linked spacer not visible in the electron density) showing the alternative recognition mode *via* the HepII O3′ and O4′ hydroxyls. The protein surface is of the PhosII-4-HepII-(1,3)-HepI structure; main chain and side chains are of both structures. *C*, HepIII-(1,2)-HepII-(1,3)-HepI-spacer (*ice blue*, HepIII not visible in the electron density), *H. influenzae* Eagan 4A Hep-anhydro Kdo (*olive green*, PDB 4E52), and *S. enterica* sv Minnesota R7 HepII-HepI-anhydro Kdo (*purple*, PDB 5OXR). The protein surface is of the HepIII-(1,2)-HepII-(1,3)-HepI structure; main chain and side chains are of all three structures. Figure created using CCP4mg.

The ligands used in these experiments are each conjugated to a chemical spacer (replacing either the core Kdo or for the HepI-(1,5)-KdoI ligand, where the lipid A GlcN would be attached to the Kdo). Electron density was only sufficiently well-defined to allow fitting of the spacer in subunit B of the HepIII-(1,2)-HepII-(1,3)-HepI (Fig. 2*B*) and PhosII-4-HepII-(1,3)-HepI (Fig. 2*D*) bound structures. The spacers do not interact directly with the coordinating protein but do form weak (3.3–3.5 Å) crystal packing and water-mediated interactions. Comparison of the spacer in the PhosII-4-HepII-(1,3)-HepI and the HepIII-(1,2)-HepII-(1,3)-HepI bound structures reveals a 90° rotation about the spacer C6–C7 bond, suggesting that there are two alternate conformations (see Figs. 3*A* and S2).

## Discussion

The work presented here seeks to build on our current understanding of innate immune recognition of LPS by hSP-D (18, 31) through further structural studies of the interaction of hSP-D with the core LPS components of the two clinically important microbial species, *H. influenzae* and *S. enterica*. The LPS ligands used in previous structural studies of recognition by hSP-D of both *H. influenzae* (18) and *S. enterica* (31) were obtained by acid hydrolysis which results in the cleavage of functional groups attached to core oligosaccharide sugars by β-elimination and reorganization of the core Kdo residue into an anhydro-furanoid derivative (18, 35). In the case of *S. enterica* (31) this β-elimination also removed KdoII and KdoIII from the LPS (see Fig. 1). While those studies revealed variable interactions of this anhydro-Kdo with the Ca1-binding pocket residues Asp325 and Arg343 and the ability of hSP-D to recognize alternative sugars when the preferred inner core is not accessible, reorganization of the Kdo and the loss of acid labile groups such as phosphates (40) precluded evaluation of hSP-D recognition of an unmodified Kdo and of the influence of key functional groups lost during hydrolysis. To address this, we used synthetic LPS components corresponding to the LPS inner core of *S. enterica*, *H. influenzae* and many other gram-negative bacteria (Fig. 1). These ligands were soaked into native rfhSP-D crystals which gave high-resolution data (1.63–1.92 Å) revealing bound ligand (Fig. 2).

In all the structures reported here, except for the HepII-(1,3)-HepI-4-PhosI LPS oligosaccharide component, ligand coordination is *via* the HepI O6′/O7′ hydroxyls to the calcium CRD and coordinating residues with no additional close, direct interactions with the protein surface. Water-mediated and crystal contacts do play a role in stabilizing the ligand within the crystal, the latter preventing ligand access to the subunit A binding site. The orientation of the bound HepI is similar to that seen for heptose-bound hSP-D (17) and in other oligosaccharide structures (18, 31). For the HepI-bound ligands which include a second, α1-3–linked, heptose HepII, this second heptose is located in approximately the same direction with respect to HepI as that seen previously for hSP-D structures that contain HepII-(α1,3)-HepI bound *via* HepI (17, 18, 31) (Fig. 3, *A* and *C*).

HepII-(1,3)-HepI–linked carbohydrates are present in many core LPS structures, suggesting a key role for hSP-D recognition of HepI, with no direct involvement of HepII, in many gram-negative bacteria. This is supported by data for the HepIII-(1,2)-HepII-(1,3)-HepI trisaccharide, a conserved core oligosaccharide backbone of *H. influenzae* LPS (Fig. 1) which suggests a clear preference for HepI recognition by hSP-D, also observed previously for *H. influenzae* strain Eagan 4A (18) and *S. enterica* Minnesota (31). While extensions to the inner core heptosyl backbone can shield the core HepI residue from recognition by rfhSP-D (18), many *H. influenzae* strains, including non-typeable *H. influenzae* strains, such as non-typeable *H. influenzae* strain 375, that are significant pathogens in children causing otitis media, sinusitis, and pneumonia (22), produce LPS molecules lacking complex extensions to their core oligosaccharide backbone. It is the recognition of these particular bacterial LPS that the HepIII-(1,2)-HepII-(1,3)-HepI–bound structure may help us understand further. Although the HepII-HepIII linkage in enterobacteria, such as *Salmonella*, is different (α1-7) to *Haemophilus*, the *S. enterica* Minnesota Rc (R5) and Rd1 (R7) mutants (Fig. 1) have been shown to be bound *via* HepI in a similar manner (31) suggesting that the nature of the HepII-HepIII linkage does not significantly influence the mode of HepI binding (see Fig. 3*C*).

For the HepI-(1,5)-KdoI ligand, representing a core LPS component of many gram-negative bacteria (Fig. 1*B*), the HepI residue is clearly present in the electron density, but there is no evidence for either the KdoI residue or the KdoI-linked spacer suggesting that there are either limited or no interactions between KdoI and hSP-D (Fig. 2*A*). This supports the binding data obtained from glycan array analysis and surface plasmon resonance which shows little difference in the binding of WT hSP-D to Hep, Hep-(1–3)-Hep, and Hep-(1–5)-Kdo (36). In the other ligand-bound structures here, the spacer is attached to the HepI where a Kdo would normally be found, but similar to KdoI of the HepI-(1,5)-KdoI ligand, the spacer makes no direct interactions with the protein surface even though different orientations of the spacer are seen for the two structures where the spacer is visible in the electron density (Fig. 3*C*). Taken together, these data suggest that where HepI is (1,5) attached to a single Kdo residue, nominally KdoI, this Kdo sugar probably does not then have a significant role in ligand recognition. However, not all bacterial LPS structures contain a single Kdo residue, for example, *S. enterica* LPS contains up to three Kdo residues (Fig. 1*F*). The question of whether the presence of these additional two Kdo residues, lost during acid hydrolysis of *S. enterica* LPS and hence not visible in the rfhSP-D LPS-bound structure (31), might lead to interactions with the protein surface remains to be answered. The HepI-(1,5)-KdoI–bound structure, where neither KdoI nor the spacer linked to KdoI where the lipid A would be attached are visible in the electron density, demonstrates how additional KdoI-linked Kdo residues, in for example Enterobacteria recognized by hSP-D (16), and the lipid A, can be accommodated in the vicinity of the binding site.

The two phosphorylated ligands used in these experiments, which correspond to the HepI-HepII backbone in *S. enterica* LPS (Fig. 1), are to the best of our knowledge the first structural definition of a human collectin molecule in complex with a phosphorylated oligosaccharide and differ only in the placement of the phosphate group, which is attached to O4′ of either HepI (HepII-(1,3)-HepI-4-PhosI) or HepII (PhosII-4-HepII-(1,3)-HepI). The HepII-(1,3)-HepI-4-PhosI LPS oligosaccharide component is the only ligand here that does not bind directly *via* HepI, recognition being achieved *via* the HepII O3′ and O4′ hydroxyls (Fig. 2*C*). It is also the only ligand here that has additional interactions with the protein *via* the binding site flanking residues Arg343 and Asp325 (Table 1; Fig. 2*C*). While the HepI O6′/O7′ hydroxyl pair on the HepII-(1,3)-HepI-4-PhosI ligand is technically available, the structure clearly shows that binding to SP-D in this manner would position the phosphate group unfavorably close to the protein surface, suggesting that when HepI is phosphorylated at O4′, binding *via* the HepI O6′/O7′ pair is no longer favorable, leading here to a reorientation of the ligand and binding *via* the HepII O3′/O4′ hydroxyls (Fig. 3*B*). The inner core oligosaccharide of *S. enterica* LPS is only phosphorylated in longer, more complex structures, such as the *S. enterica* serovar Minnesota Ra phenotype (41, 42) shown in Figure 1*F*, which is not recognized by hSP-D (16). This is illustrated by the structures presented here, phosphorylation of both HepI and HepII at O4′ will prevent O6′/O7′ recognition and the HepII O3′ pentasaccharide extension Glc-(Gal)Gal-Glc(GlcNAc) (43) will prevent the alternative recognition *via* O3′/O4′. In the LPS from Rc (R5) and Rd1 (R7) mutants, which are bound by hSP-D (16, 31), these phosphate substitutions are not present (Fig. 1*F*). It is not clear why, in the presence of phosphorylation of both HepI and HepII, either O6′/O7′ or O3′/O4′ on HepIII are not utilized for either the *H. influenzae* strain Eagan WT LPS (Fig. 1*E*) or the *S. enterica* serovar Minnesota Ra phenotype LPS (Fig. 1*F*) which are both reported to exhibit minimal binding by hSP-D (16, 18).

The structures presented here show that inner core HepI binding is the preferred mode of recognition with these small synthetic core oligosaccharides revealing no direct interactions between the protein and either an α1-3 linked HepII or KdoI. They also highlight the importance of Hep phosphorylation in both SP-D recognition and evasion, with phosphorylation of HepI at O4′ sterically preventing the preferred HepI O6′/O7′ binding, which is then overcome here by alternate recognition of the α1-3–linked HepII O3′/O4′ hydroxyl pair (Fig. 3*B*). Other alternative modes of recognition, such as the terminal Glc recognition of *S. enterica* Minnesota R5 (31), may also be adopted. Flexibility in LPS recognition appears to be a key attribute of the hSP-D protein. Previously, binding data from biochemical analyses and crystal structures have demonstrated that carbohydrate extensions to the core oligosaccharide can effectively shield inner core residues, for example HepI, from recognition by hSP-D (18). The structures presented here build on this concept by demonstrating that recognition is also significantly affected by the phosphorylation of specific residues in the core LPS oligosaccharide. LPS phosphorylation is a consistent trend across gram-negative bacteria and likely provides an additional mechanism by which bacteria avoid detection by immune proteins, including hSP-D, that are encountered during their commensal and disease-causing lifestyles within the human host.

## Experimental procedures

### Cloning, expression, and purification of rfhSP-D

The rfhSP-D protein was expressed in *Escherichia coli* using a bacterial expression system, as detailed by Madan *et al.*, 2001 (44) and Strong *et al.*, 2002 (19). Each chain contains 177 amino acids corresponding to residues Gly179 to Phe355, comprising a short (8 x Gly-Xaa-Yaa) collagen-like domain, an α-helical coiled-coil neck domain, followed by the CRD. The rfhSP-D was purified by a procedure involving denaturation and refolding of the inclusion bodies, followed by ManAc affinity and gel filtration chromatography. The rfhSP-D was passed through a polymyxin B column (Detoxi-Gel, Pierce) to remove endotoxin resulting in an endotoxin level of less than 10 pg/μg protein.

### Oligosaccharide synthesis

Four synthetic oligosaccharides were repurposed for use in these experiments. The complete synthesis for three of these oligosaccharides, HepIII-(1,2)-HepII-(1,3)-HepI; HepII-(1,3)-HepI-4-PhosI; and PhosII-4-HepII-(1,3)-HepI have been reported in the literature (37, 39). The report of the synthesis of the Hep-Kdo disaccharide (HepI-(1,5)-KdoI) ligand (38) is not routinely available in the literature and we have included the synthesis of this ligand in the Supplementary Material.

### Crystallization and data collection

Native crystals of rfhSP-D were grown in sitting drops consisting of an equal volume of protein solution (1 μl) and precipitant solution, consisting of 16% PEG 4000 and 0.1 M Tris pH 8.0. Crystals were prepared for cryocooling using 2-methyl-2,4-pentanediol (MPD) in precipitant buffer. Ligand was introduced into the crystal by the addition of ligand to the cryobuffer. Successful additions of 2 to 3 μl aliquots of the increasing concentrations (5–20%) of MPD cryobuffer were added to each well, followed by the addition of a further 2 μl aliquot of 20% MPD cryobuffer with a final exchange of 8 μl of the microbridge solution with 20% MPD cryobuffer. The concentration of each ligand in the respective cryobuffer was 20 mM, apart from the HepI-(1,5)-KdoI ligand, which was at a final concentration of 33.75 mM. Data were collected at Diamond Light Source stations I03 and I04, from a single crystal in each case, using a Pilatus3 6M detector. Integrated intensities were calculated using *Mosflm* (45) and data was processed using the *Aimless*, *Truncate*, *Uniquify*, and *Sortmtz* programmes as part of the CCP4 programme suite (46). Data collection and processing statistics are given in Table 2.

**Table 2:** Crystallographic data and refinement statistics

	HepI-Kdo–bound rfhSP-D			HepIII-HepII-HepI–bound rfhSP-D			PhosII-HepII-HepI–bound rfhSP-D			HepII-HepI-PhosI–bound rfhSP-D		
Data collection												
Synchrotron station	DLS I04-1			DLS I03			DLS I03			DLS I03		
Wavelength (Å)	0.920			0.9762			0.9762			0.9762		
Space group	P 2_1_			P 2_1_			P 2_1_			P 2_1_		
Cell dimensions (Å, °)	a = 55.18			a = 55.24			a = 55.13			a = 55.44		
	b = 106.78			b = 107.56			b = 106.25			b = 108.18		
	c = 55.47			c = 55.56			c = 55.34			c = 55.65		
	α = 90			α = 90			α = 90			α = 90		
	β = 92.25			β = 90.84			β = 91.13			β = 90.90		
	γ = 90			γ = 90			γ = 90			γ = 90		
Resolution range (Å)	55.43–1.75 (1.78–1.75)			55.55–1.63 (1.66–1.63)			53.13–1.85 (1.89–1.85)			55.64–1.92 (1.97–1.92)		
Observations	223,189 (12,162)			206,299 (10,173)			133,717 (8485)			130,259 (8221)		
Unique reflections	63,620 (3472)			79,745 (3899)			53,663 (3311)			49,393 (3267)		
Completeness (%)	98.1 (97.3)			98.8 (98.2)			98.8 (98.9)			98.8 (98.0)		
R_merge_^a^	0.051 (0.366)			0.051 (0.345)			0.061 (0.333)			0.078 (0.305)		
CC1/2^b^	0.998 (0.753)			0.978 (0.752)			0.990 (0.674)			0.973 (0.788)		
Mean (I/σ(I))	11.9 (3.0)			9.9 (2.9)			9.2 (3.0)			8.0 (3.0)		
Refinement												
Protein atoms												
Residues (Chain A)	151			147			149			147		
Residues (Chain B)	152			147			150			147		
Residues (Chain C)	150			146			150			145		
Water molecules	385			371			303			335		
Other molecules												
Subunit	A	B	C	A	B	C	A	B	C	A	B	C
Calcium ions	3	3	3	3	3	3	3	3	3	3	3	3
Ligand	-	1	1	-	1	1	-	1	1	-	1	1
R_work_^c^ (%)	17.0			17.7			17.8			18.1		
R_free_^d^ (%)	20.2			19.9			20.1			21.2		
Bond length r.m.s.d (Å)	0.015			0.023			0.022			0.015		
Bond angles r.m.s.d (°)	1.841			1.842			1.902			1.815		
Average B-Values (Å^2^)												
Protein	25.5			22.3			25.9			23.8		
Water	32.8			31.3			32.8			30.1		
Ligands	33.5			29.7			23.7			27.8		
PDB ID	9QVU			9QW3			9QW4			9QW2		
Ramachandran plot values (%)^e^												
Favored	97.54			98.16			98.42			97.92		
Allowed	2.46			1.84			1.58			2.08		
Outliers	0.00			0.00			0.00			0.00		

Figures in parentheses refer to the highest resolution bin.

a R_merge_ = Σ_h_Σ_j_ | I_h,j_ – I_h_ |/Σ_h_Σ_j_ | I_h,j_|, where I_h,j_ is the j^th^ observation of reflection *h* and I_h_ is the mean of the *j* measurements of reflection h.

b CC1/2 correlation coefficient between random half-sets of data.

c R_work_ = Σ_h_ | |F_oh_| - |F_ch_| |/Σ_h_ |F_oh_|, where F_oh_ and F_ch_ are the observed and calculated structure factor amplitudes, respectively, for reflection *h*.

d R_free_ is equivalent to R_work_ for a randomly selected subset (5%) of reflections not used in the refinement.

e Determined according to MolProbity.

### Structure solution and refinement

Isomorphism was sufficient to allow the atomic coordinates of the published 1.6 Å native rfhSP-D crystal structure (PDB:1PW9, (27)) to be used as a starting model for the rfhSP-D ligand-bound structures. Initial models were built in *Coot* (47) after rigid body refinement with *Refmac5* (48). Subsequent model building was completed over multiple rounds of restrained refinement using *Refmac5* alternated with rounds of manual model building with *Coot*. Ligand coordinates were imported *via Coot* from the Protein Data Bank (PDB) with the exception of the two chemical spacers, which were generated manually using *AceDRG* (49), which was also used to generate the dictionaries for the two chemical spacers. The quality of the final model was verified using the *MolProbity* server and *Privateer* as part of the CCP4i2 suite (50, 51) as well as the PDB validation software. Final refinement statistics are provided in Table 2. Molecular figures were generated using CCP4mg (52).

## Data availability

The coordinates and structure factors for the HepI-(1,5)-KdoI (9QVU), HepIII-(1,2)-HepII-(1,3)-HepI (9QW3), HepII-(1,3)-HepI-4-PhosI (9QW2), and PhosII-4-HepII-(1,3)-HepI (9QW4) ligand-bound structures have been deposited with the PDB alongside the coordinates and dictionaries for the two chemical spacers. The raw experimental diffraction image data are available *via* Keele University Data Repository (DOI: https://doi.org/10.21252/yzsx-0e16; DOI: https://doi.org/10.21252/nd1w-be36; DOI: https://doi.org/10.21252/m42p-9x07; DOI: https://doi.org/10.21252/bnzw-6034).

## Supporting information

This article contains supporting information (53, 54).

## Conflicts of interest

The authors declare that they have no conflicts of interests with the contents of this article.
