# Supplementary material for: Phosphorylation of inner core heptose is a major determinant of bacterial surface lipopolysaccharide recognition by the innate immune protein hSP-D
Source: J Biol Chem. 2026 Feb 20;302(4):111307. doi: 10.1016/j.jbc.2026.111307 (PMC13015573; doi:10.1016/j.jbc.2026.111307)
Supplement: Supplementary material [file mmc1.pdf]

SUPPLEMENTARY MATERIAL

Phosphorylation of inner core heptose is a major determinant of bacterial surface lipopolysaccharide recognition by the innate immune protein hSP-D

Harry M. Williams, Alastair Watson, Jens Madsen, Howard W. Clark, Derek W. Hood, Stefan Oscarson, Trevor J. Greenhough and Annette K. Shrive

Table S1. Calcium-Protein/Water (W) Bond Lengths (Å).

Table S2. Interactions between ligands and residues in symmetry-related molecules (Å).

Figure S1. The rfhSP-D trimer with bound HepIII-HepII-HepI

Figure S2. Schematic diagrams of the bound ligands showing the protein-ligand interactions.

Synthesis of the HepI-(1,5)-KdoI disaccharide

Table S1. Calcium-Protein/Water (W) Bond Lengths (Å).

Atom 1	Atom 2		HepI-Kdo			HepIII-HepII-HepI			PhosII-HepII-HepI			HepII-HepI-PhosI		
			A	B	C	A	B	C	A	B	C	A	B	C
Ca1	Glu321	OE1	2.58	2.55	2.48	2.57	2.52	2.63	2.50	2.50	2.73	2.56	2.48	2.68
	Asn323	OD1	2.44	2.43	2.40	2.36	2.35	2.37	2.45	2.35	2.47	2.41	2.53	2.51
	Glu329	OE1	2.50	2.40	2.36	2.43	2.42	2.42	2.38	2.31	2.38	2.35	2.41	2.39
	Asn341	OD1	2.39	2.44	2.40	2.36	2.39	2.39	2.40	2.34	2.43	2.34	2.37	2.36
	Asp342	O	2.59	2.51	2.58	2.51	2.52	2.54	2.57	2.60	2.52	2.55	2.57	2.53
	Asp342	OD1	2.40	2.35	2.32	2.40	2.32	2.32	2.39	2.21	2.35	2.41	2.30	2.40
Ca2	Asp297	OD1	2.66	2.58	2.64	2.55	2.62	2.59	2.62	2.67	2.76	2.62	2.66	2.63
	Asp297	OD2	2.42	2.42	2.58	2.44	2.44	2.43	2.38	2.59	2.37	2.40	2.48	2.40
	Glu301	OE1	2.50	2.47	2.47	2.40	2.47	2.45	2.50	2.42	2.55	2.52	2.53	2.57
	Glu301	OE2	2.56	2.52	2.55	2.47	2.48	2.48	2.49	2.55	2.54	2.47	2.53	2.51
	Asn324	OD1	2.52	2.58	2.59	2.55	2.65	2.56	2.60	2.44	2.54	2.51	2.48	2.54
	Glu329	O	2.46	2.45	2.39	2.42	2.48	2.43	2.43	2.38	2.37	2.47	2.40	2.37
	Asp330	OD1	2.45	2.43	2.36	2.44	2.38	2.43	2.38	2.39	2.36	2.32	2.31	2.38
	W		2.47	2.32	2.42	2.45	2.35	2.33	2.37	2.28	2.28	2.47	2.35	2.38
Ca3	Glu301	OE1	2.28	2.40	2.36	2.42	2.37	2.37	2.32	2.35	2.46	2.28	2.46	2.31
	Asp330	OD1	2.50	2.57	2.58	2.54	2.52	2.57	2.60	2.48	2.60	2.56	2.54	2.60
	Asp330	OD2	2.46	2.51	2.48	2.45	2.50	2.48	2.50	2.53	2.47	2.42	2.40	2.52
	W		2.30	2.18	2.24	2.20	2.26	2.33	2.14	2.27	2.25	2.00	2.29	2.23
	W		2.36	2.37	2.38	2.29	2.38	2.35	2.27	2.35	2.31	2.27	2.27	2.26
	W		2.38	2.37	2.45	2.36	2.40	2.35	2.32	2.40	2.37	2.38	2.32	2.35
	W		2.58	2.38	2.45	2.37	2.40	2.38	2.41	2.43	2.46	2.51	2.51	2.39

Table S2. Interactions between ligands and residues in symmetry-related molecules (Å).

Atom 1	Atom 2	HepI-Kdo			HepIII-HepII-HepI			PhosII-HepII-HepI			HepII-HepI-PhosI		
		A	B	C	A	B	C	A	B	C	A	B	C
HepI	O2′ Ser226/A OG	-	-	-	-	-	2.85	-	-	2.91	-	-	-
HepI	O2′ Ser226/C OG	-	3.00	-	-	3.05	-	-	-	-	-	-	-
HepII	O4′ Lys229/A NZ	-	-	-	-		2.54	-	-	-	-	-	-
HepII	O4′ Lys229/C NZ	-	-	-	-	2.61	-	-	-	-	-	-	-
HepII	O7′ Ser226/C O	-	-	-	-	-	-	-	2.76	-	-	-	-
	O7′ Ser226/C OG	-	-	-	-	-	-	-	3.09	-	-	-	-
HepII	O6′ Tyr228/B OH	-	-	-	-	-	-	-	3.17	-	-	-	-
HepII/Phos	O1′ Tyr228/B OH	-	-	-	-	-	-	-	2.59	-	-	-	-
	O1′ Ser239/C OG	-	-	-	-	-	-	-	2.62	-	-	-	-
HepII/Phos	O2′ Lys229/C NZ	-	-	-	-	-	-	-	2.91	-	-	-	-

Figure S1. The rfhSP-D trimer with bound HepIII-HepII-HepI.

The rfhSP-D trimer from the HepIII-(1,2)-HepII-(1,3)-HepI ligand-bound crystal structure is shown. Each protomer also binds three calcium ions, which are represented as green spheres. In subunits B (Gold) and C (Orange), where the HepIII-(1,2)-HepII-(1,3)-HepI ligand is bound, the ligand is shown at the Ca1 site in yellow. Note that in subunit B the HepI-linked spacer is visible in the map and was therefore fitted in the structure, in subunit C the spacer is missing. HepIII is not visible in the electron density in either subunit. Image created using CCP4mg.

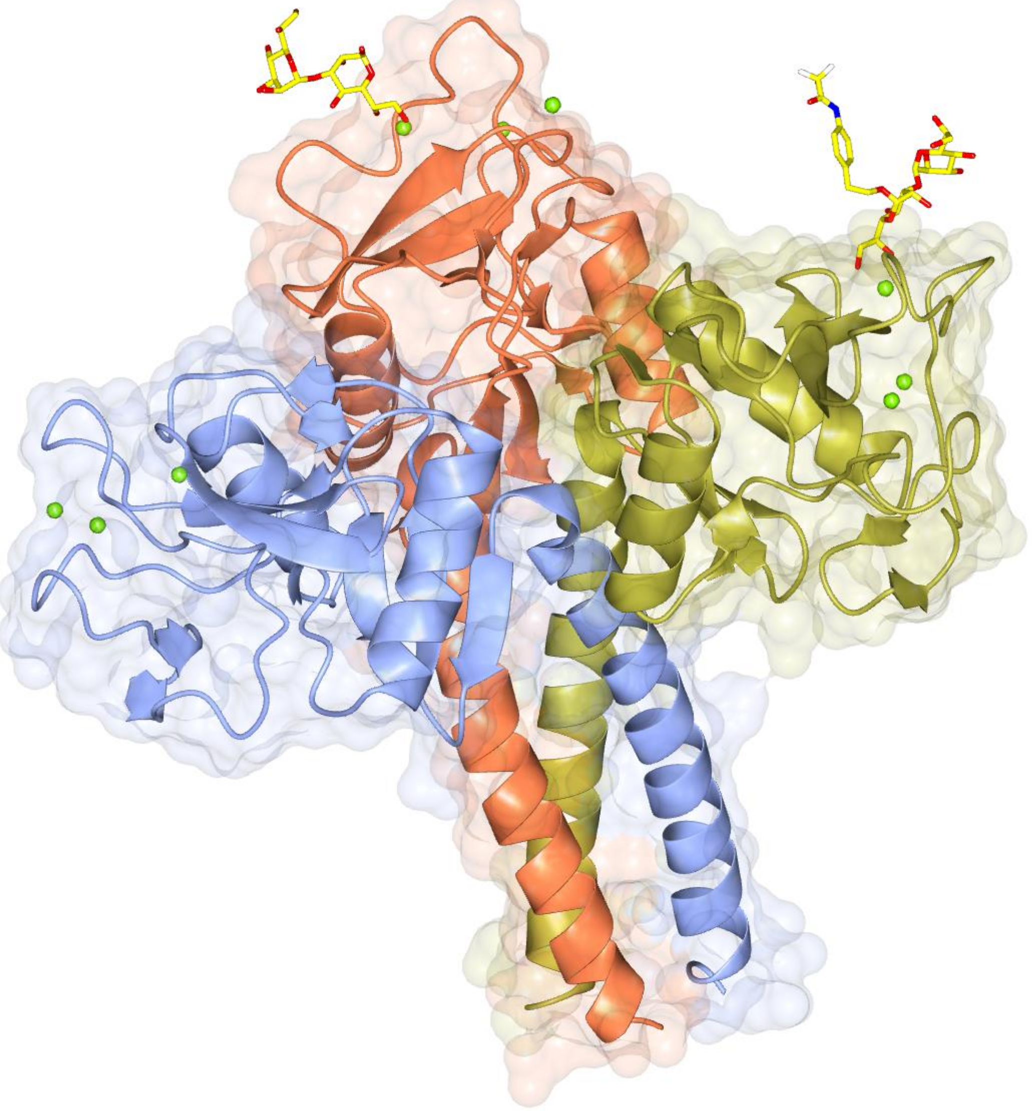

Figure S2. Schematic diagrams of the bound ligands showing the protein-ligand interactions.

(**A**) HepI-(1,5)-KdoI. Only HepI, coordinated to Ca1 by the O6′ and O7′ hydroxyls, is visible in the electron density (**B**) HepIII-(1,2)-HepII-(1,3)-HepI with HepI, HepII and the spacer visible in the electron density and coordination to Ca1 via the HepI O6′ and O7′ hydroxyls (**C**) HepII-(1,3)-HepI-4-PhosI showing the alternative mode of recognition via the HepII O3′ and O4′ hydroxyls of HepII. The spacer linked to HepI is not visible in the electron density. (**D**) PhosII-4-HepII-(1,3)-HepI, coordinated to Ca1 by the HepI O6′ and O7′ hydroxyls. Figure assembled using modified diagrams created using the EMBL-EBI LIGPLOT+ programme.

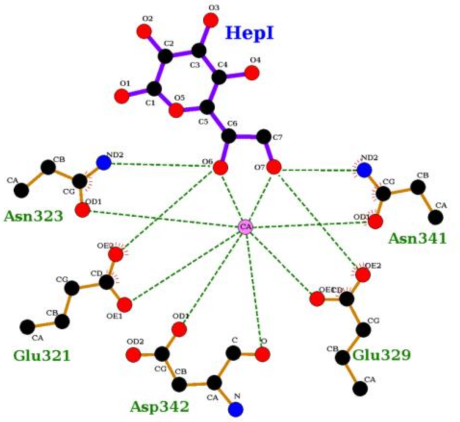

A. HepI-KdoI

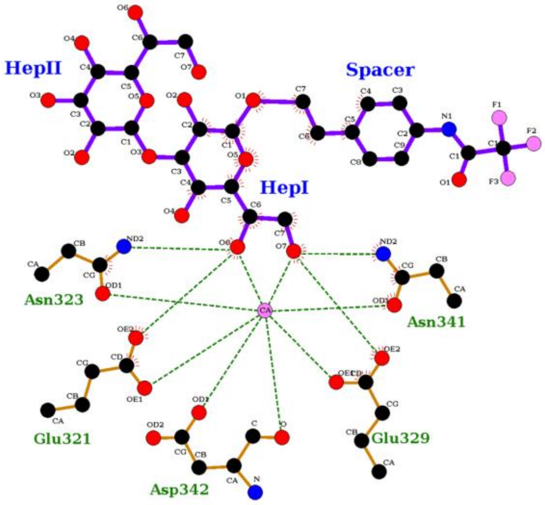

B. HepIII-(1,2)-HepII-(1,3)-HepI

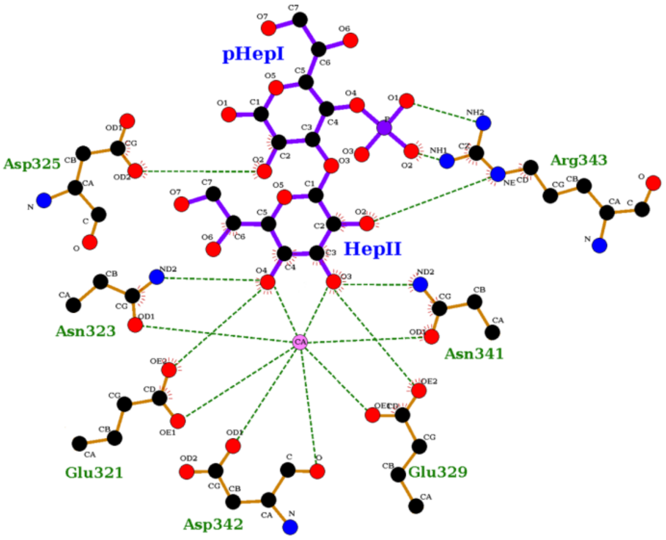

C. HepII-(1,3)-Hep1-4-PhosI

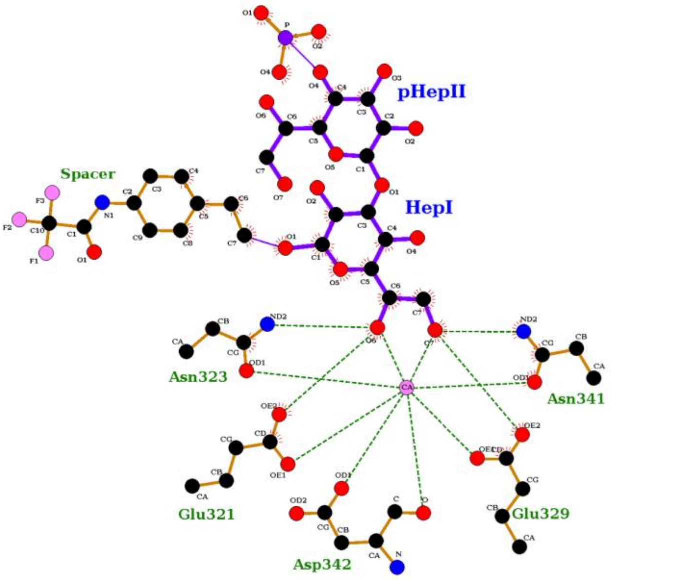

D. PhosII-4-HepII-(1,3)-HepI

Synthesis of the HepI-(1,5)-KdoI disaccharide

The synthesis of the HepI-(1,5)-KdoI disaccharide (Scheme 1) was reported in the PhD thesis "Synthesis of oligosaccharides corresponding to structures found in the cell walls of *Salmonella typhimurium* and *Moraxella catarrhalis*" of Kerstin Ekelöf from Stockholm University in 1996.

In short, the spacer-equipped 5-OH Kdo acceptor **1** **[**Ref. 1] was coupled with the perbenzoylated L-*glycero*-D-*manno*-heptopyranosyl bromide donor **2** [Ref. 2] using silver triflate as promoter to afford the disaccharide **3**. Deprotection was performed in four steps as described for a similar structure in Ref. 1: first acid hydrolysis, to remove the isopropylidene group, then Zemplen deacylation, to remove benzoyl esters, followed by catalytic hydrogenolysis, to remove the benzyl ether, and finally saponification, to hydrolyse the methyl ester and the trifluoroacetamide, to afford the target structure **4** (HepI-(1,5)-KdoI).

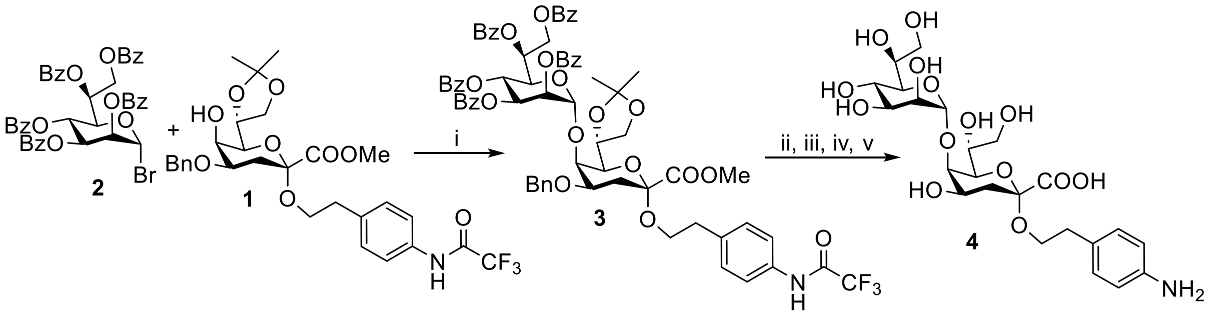

Scheme 1: Synthesis of disaccharide HepI-(1,5)-KdoI (**4**). Reagents: i) AgOTf, collidine, CH_2Cl_2; ii) CF_3COOH (90% aq.); iii) NaOMe, MeOH; iv) H2, Pd/C, MeOH/HOAc; v) NaOH, H_2O/MeOH.

References:

1. Synthesis of 2-(4-aminophenyl)ethyl 5-*O*-(3,4,6-tri-*O*-β-D-glucopyranosyl-α-D-glucopyranosyl)-α-D-*manno*-2-octulopyranoside, a highly branched pentasaccharide corresponding to structures found in lipopolysaccharides from *Moraxella catarrhalis,* K. Ekelöf and S. Oscarson, *Carbohydr. Res.*, 278 (1995) 289-300.
2. Synthesis of 2-(*p*-trifluoroacetamidophenyl)ethyl 3-*O*-[7-*O*-(L-*glycero*-α-D-*manno*-heptopyranosyl)-L-*glycero*-α-D-*manno*-heptopyranosyl]-L-*glycero*-α-D-*manno*-heptopyranoside, corresponding to the heptose part of the *Salmonella* Ra core saccharide, P. J. Garegg, S. Oscarson, H. Ritzén, and M. Szönyi, *Carbohydr. Res.*, 228 (1992) 121-128.
